# Prevalence and decay of maternal pneumococcal and meningococcal antibodies: A meta-analysis of type-specific decay rates

**DOI:** 10.1016/j.vaccine.2017.09.002

**Published:** 2017-10-13

**Authors:** Merryn Voysey, Andrew J. Pollard, Manish Sadarangani, Thomas R. Fanshawe

**Affiliations:** a Nuffield Department of Primary Care Health Sciences, University of Oxford, Oxford, UK; b Oxford Vaccine Group, Department of Paediatrics, University of Oxford, and NIHR Oxford Biomedical Research Centre, Oxford, UK; c Vaccine Evaluation Center, BC Children’s Hospital Research Institute, University of British Columbia, Vancouver, Canada

**Keywords:** Maternal antibody, Half-life, Transplacental, Pneumococcal, Meningococcal

## Abstract

**Background:**

At the time of an infant’s initial vaccination at age ∼2 to 3 months, some infants already have maternal antibodies against vaccine antigens and these can suppress the immune response to vaccination. Modelling the effects of maternal antibody and the timing of infant doses on the antibody response to vaccination, requires estimates of the rate of maternal antibody decay. Decay rates are not well characterised in the medical literature. We investigated variation in the prevalence of maternal anti-capsular pneumococcal and meningococcal antibodies in infants in 14 countries, and estimated type-specific half-lives.

**Methods:**

Individual participant serological data were obtained from clinical trials. Half-lives were estimated from antibody concentrations in infants who did not receive meningococcal or pneumococcal vaccines.

**Results:**

The seroprevalence of maternal pneumococcal antibodies was highest for serotypes 14, and 19F (92% and 80% respectively) and lowest for serotypes 4 and 1 (30% and 34% respectively). Half-life estimates ranged from 38.7 days (95% CI 36.6–41.0) for serotype 6B, to 48.3 days (95% CI 46.7–50.2) for serotype 5. The overall half-life was 42.6 days (95% CI 41.5–43.7). Seroprevalence was highest in Mali, Nigeria, India, and the Philippines, (all >65%) and lowest in the Czech Republic and Finland (both <45%).

In studies of meningococcal vaccines, seroprevalence was 13% for group C (half-life 39.8 days, 95% CI 33.4–49.4) and 43% for group A (half-life 43.1 days 95% CI 39.8–47.2).

**Conclusion:**

Substantial proportions of infants in many countries have antibodies to vaccine serotypes of pneumococcus, however fewer infants have maternally acquired antibodies to groups A and C meningococcus.

Passively-acquired antibodies to capsular polysaccharides decay with a half-life of approximately 6 weeks. These estimates are useful for modelling the impact of proposed vaccination programmes, and consideration of schedules with a delayed start.

## 1 Background

*Streptococcus pneumoniae* and *Neisseria meningitidis* are leading causes of severe bacterial infection in infants and adults worldwide. Administration of licenced conjugate-polysaccharide vaccines in infants is effective in preventing vaccine-type disease, and also prevents asymptomatic carriage of the bacteria in the nasopharynx of healthy individuals, which in turn induces herd protection.

At the time of their first vaccination, some infants already have antibodies against vaccine types of pneumococci and meningococci due to placental transfer of antibody in pregnancy. Almost all antibody is transferred across the placenta in the third trimester of pregnancy and if still present at the time of vaccination, this passively-acquired antibody can interfere with the infant’s ability to mount a robust immune response to vaccination [1]. The concentration of antibody in infant sera at the time of their first vaccination depends on the serum antibody concentration in the mother, placental function, and the gestational age at birth. In addition, differences may exist between species or between serotypes in the rate of transfer of antibody across the placenta, and the rate of decay of antibody between birth and the first vaccination, all of which will affect the residual concentration of antibody circulating at the time of vaccination.

We previously modelled the effects of maternal antibody and age at first vaccination on infant immune responses, and computed the effect of delaying the first dose of vaccine. Such computations require estimates of the rate of decay of maternal antibodies, however reliable estimates of half-lives in the medical literature were found to be scarce.

The half-life of passively-acquired maternal pneumococcal antibodies has been estimated as approximately 35 days. This estimate comes from a combined analysis of antibody against two serotypes of pneumococcus in one study in Bangladesh [2]. O’Dempsey et al. describe a half-life of 20–30 days for maternal pneumococcal antibodies. and 20 days for maternal meningococcal antibodies [3]. In contrast, Shahid et al. estimated the half-life of maternal meningococcal antibodies to be 45 days in Bangladesh [4]. These estimates from studies conducted almost two decades ago have not been confirmed in other studies and the variability in these half-lives is unknown. It is also unclear whether they apply to children in different geographic regions or whether antibodies against different polysaccharides decay at different rates.

We investigated variation in the prevalence of maternal pneumococcal and meningococcal antibodies in infants, and estimated the type-specific half-lives of passively-acquired antibodies in sera from children who had not received pneumococcal or meningococcal vaccines in order to inform consideration of delayed start vaccination and modelling of the interplay between maternal and infant immunisation programmes.

## 2 Methods

### 2.1 Data

Fully anonymised data from clinical trials were available through https://clinicalstudydatarequest.com/. Studies were selected from all infant pneumococcal conjugate vaccine (PCV) and meningococcal vaccine trials listed on the website which were described as having individual participant data available. All trials were conducted by a single manufacturer (GlaxoSmithKline) with assays conducted in a single central laboratory as this was the only manufacturer with suitable data available from infant vaccine trials.

### 2.2 Seroprevalence data

For the assessment of the prevalence of maternal antibody, studies were included in which blood samples were taken prior to receipt of the first dose of vaccine and the concentration of type-specific pneumococcal or meningococcal IgG was measured.

### 2.3 Antibody half-life data

For the assessment of antibody half-lives, data were included from a subset of studies in which antibody concentrations were measured at two time points and some children were allocated to unvaccinated control groups. Whilst included infants did not receive pneumococcal or meningococcal vaccines, they still received three doses of other routine vaccines according to the local schedule. The first time point was therefore prior to receipt of any vaccine (at study enrolment), and the second time point was one month after the third dose of routine (non-study) infant vaccines (at age ∼5 to 7 months).

Children were included in the analysis as follows:

Pneumococcal vaccine studies:

• Studies of PCV7 versus no PCV: Antibody concentrations from children in the ‘no PCV’ control group were included
• Studies of PCV7 versus either PCV10 or PCV11: Antibody concentrations for pneumococcal serotypes 1, 5 and 7F (non-PCV7 serotypes) were included from children in the PCV7 group.

Meningococcal vaccine studies:

• Studies of group C meningococcal (MenC) vaccine versus no MenC vaccine: Antibody concentrations from children in the ‘no MenC’ control group were included
• Studies comparing multivalent meningococcal vaccines (AC or ACWY) versus MenC vaccine: Antibody concentrations for group A were included from children who received MenC vaccine. No data were available for capsular groups W and Y.

Due to some studies being conducted in multiple countries, children were grouped according to country within study (‘country cohorts’) for the analysis.

Samples from some children were excluded from the analysis of half-lives. Firstly, children were excluded from the analysis if their initial antibody concentration was below the limit of quantification of the assay (LLQ) as these results give half-life estimates which are undefined. Secondly, children were excluded if their antibody concentration increased between the first and second time points, as such results give negative half-lives.

Thirdly, children were excluded if their antibody concentrations exhibited an implausibly slow rate of decay during the time between samples as these results were thought to be influenced by nasopharyngeal carriage or unreported vaccinations. An arbitrary cut-off of a half-life of 80 days was used to make such exclusions as this is more than twice the half-life reported previously for pneumococcal antibodies and for many other antigens.[2], [3], [5], [6], [7] Infants with antibody below the LLQ at the second time point were retained in the analysis and these values were substituted with half the lower limit for analysis purposes. The rate of decay calculated for these infants is therefore an approximation.

### 2.4 Maternal antibody seropositivity in infants

Infant seropositivity to maternal antibody at the first visit (baseline) was defined as an antibody concentration above the LLQ at baseline, prior to receipt of any meningococcal or pneumococcal vaccination.

### 2.5 Half-life estimation

Antibody concentrations were log_2_-transformed and assumed to decay log-linearly. For each individual, the rate of decay per day was computed as the difference between the log_2_-antibody concentrations at the two time points divided by the number of days between the two samples.

All individual serotype-specific pneumococcal antibody decay rates from participants in all country cohorts were combined in a single mixed effects model with child-specific random intercepts. The model included fixed effects for country cohort and serotype. The serotype-specific decay rates were obtained from the model coefficients and the overall decay rate computed as a linear combination of the serotype-specific fixed effects. Decay rates were converted into half-lives by taking the reciprocal of the absolute value of the decay rate.

Heterogeneity in decay rates across country cohorts and across serotypes was assessed using an F-test (type III p value).

The assumption that passive antibody decays in a log-linear (exponential) fashion was tested in a separate model by including the age of the child at the time of the first vaccination.

Analysis of groups A and C meningococcal antibodies was similar but models contained no terms for capsular group or participant as each participant only contributed once to the analysis. Due to the limited data available for these antibodies, the two capsular groups were analysed separately.

To examine the influence of including studies which only contributed to the analysis of a small number of serotypes, a sensitivity analysis was conducted for pneumococcal serotype-specific half-lives, which included only studies which measured the majority of serotypes, i.e. the serotypes contained in the PCV7 vaccine.

Analyses were conducted using SAS version 9.4 and R version 3.3.2.

## 3 Results

### 3.1 Seroprevalence of maternal antibodies

5097 children in 16 cohorts of children from 13 countries had pneumococcal antibody concentrations assessed prior to the first dose of vaccine, and 2925 infants from 5 cohorts in 4 countries had meningococcal antibody concentrations available (Table S1 and S2). Children ranged in age from 5 weeks to 23 weeks at the time of their first vaccination and were from a diverse group of countries in Europe, Africa, Latin America, and South and East Asia.

The seroprevalence of maternal antibodies in infants was highest for serotypes 14, and 19F (92% and 80% respectively) and lowest for serotypes 4 and 1 (30% and 34% respectively) (Fig. 1A). Overall across serotypes, seroprevalence was highest in studies in which children were younger (Mali, Nigeria, India, and The Philippines, (all >65%)) and lowest in studies of older children (The Czech Republic and Finland (both <45%)) (Table 1, Table S1).

**Fig. 1A:**
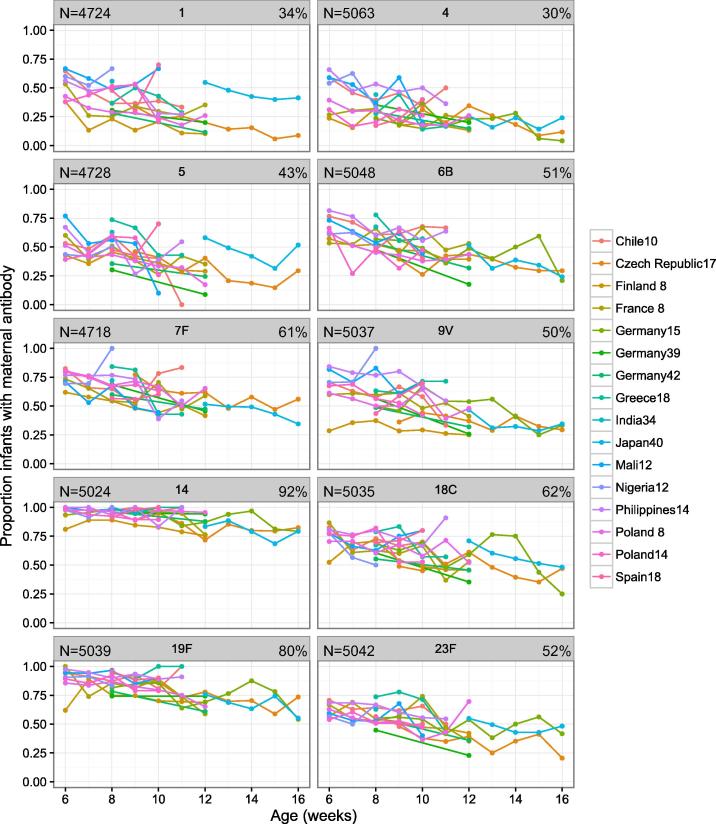
Proportion of infants in each cohort with detectable levels of serotype-specific pneumococcal maternal antibody prior to their first vaccination, according to age at enrolment. Each dot indicates the cross-sectional proportion of children within each country cohort and each week of age, who had detectable levels of serotype-specific maternal pneumococcal antibody prior to the first dose of vaccine. The number of infants included in the analysis is displayed in the top left hand corner of each cell, and the percentage of infants with maternal antibody is displayed in the top right hand corner of each cell.

**Table 1:** Percentage of infants with detectable levels of maternal antibody prior to first vaccination.

Country Cohort	Number of participants with data at baseline	Number of antibody concentration measures at baseline (all serotypes, capsular groups)	% above the LLQ	
Pneumococcal antibody data				
Czech Rep 17	449	4406	44.2%	
Finland 8	835	8332	44.4%	
Germany 39	132	1312	48.1%	
Japan 40	359	3552	48.5%	
Germany 42	677	6661	51.3%	
France 8	212	2091	56.0%	
Germany 15	336	2319	57.0%	
Spain 18	85	846	58.9%	
Poland 8	599	5946	59.2%	
Poland 14	203	2030	60.0%	
Greece 18	63	622	63.3%	
Chile 10	235	2305	64.2%	
Nigeria 12	119	1185	67.8%	
Mali 12	233	2257	67.9%	
Philippines 14	200	1998	71.9%	
India 34	360	3596	76.5%	

Meningococcal antibody data			**Group A**	**Group C**
Thailand 43	850	1550	38.6%	8.2%
Poland 13	396	396	NA	8.9%
Philippines 44	727	1422	47.5%	11.4%
Spain 22	465	465	NA	19.1%
Spain 27	459	459	NA	21.6%

Meningococcal antibodies were measured in studies of children aged 6–12 weeks at the time of the first vaccination. 374/2889 (13%) infants had detectable levels of group C and 604/1403 (43%) had group A meningococcal antibodies prior to vaccination. (Table 1, Fig. 1B)

**Fig. 1B:**
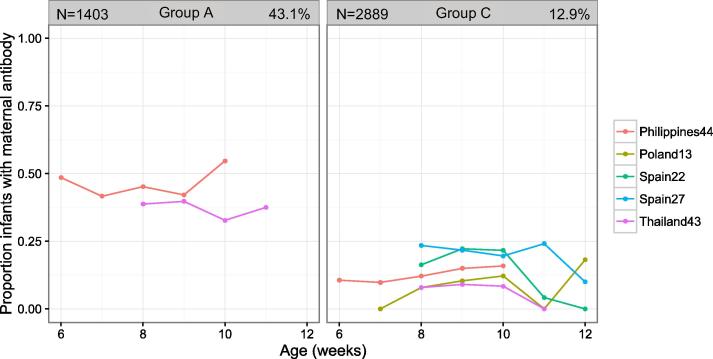
Proportion of infants in each cohort with detectable levels of group-specific meningococcal maternal antibody prior to their first vaccination, according to age at enrolment. Each dot indicates the cross-sectional proportion of children within each country cohort and each week of age, who had detectable levels of serotype-specific maternal meningococcal antibody prior to the first dose of vaccine. The number of infants included in the analysis is displayed in the top left hand corner of each cell, and the percentage of infants with maternal antibody is displayed in the top right hand corner of each cell.

### 3.2 Half-lives

#### 3.2.1 Pneumococcal antibodies

In studies of PCVs there were 10 of 16 country cohorts in which a second blood sample was taken from infants 3–5 months after the first. Of these, 701 infants had 1917 pairs of pneumococcal antibody measures available for the analysis of half-lives (Fig. 2A).

**Fig. 2A:**
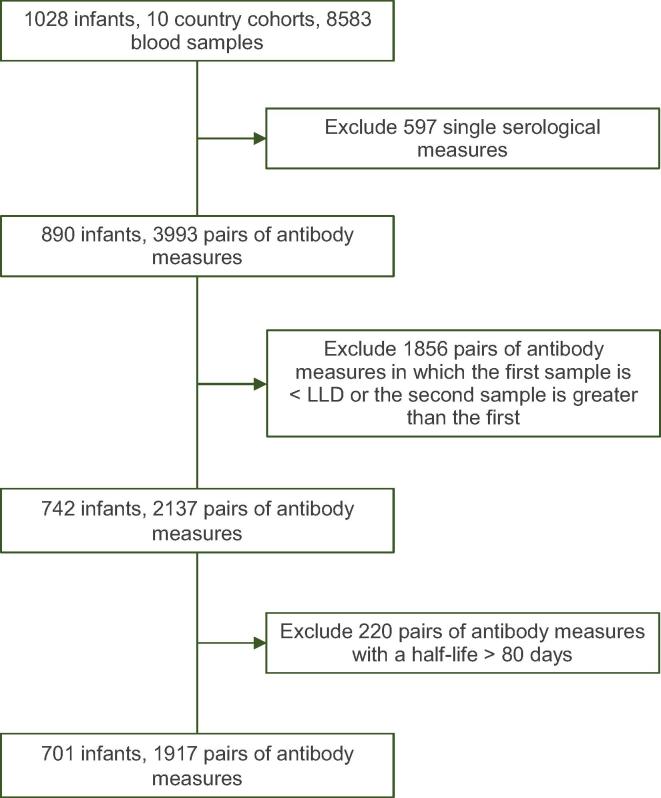
Flow chart of available data and reasons for exclusions from half-life estimations for pneumococcal immunoglobulin G concentrations. LLQ: Lower limit of assay quantification.

For each serotype, between 3 and 9 country cohorts had sufficient data to be included in the meta-analysis. Serotype 4 had the least number of samples available for analysis (N = 86) and serotype 7F had the most (N = 409) (Fig. 3, Fig. 4A).

**Fig. 2B:**
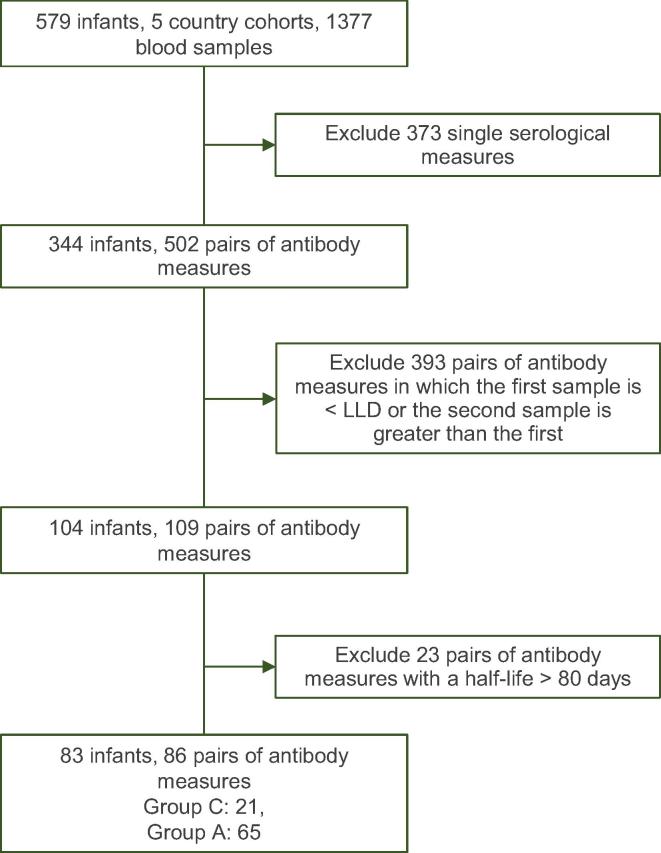
Flow chart of available data and reasons for exclusions from half-life estimations for meningococcal immunoglobulin G concentrations. LLQ: Lower limit of assay quantification.

**Fig. 3:**
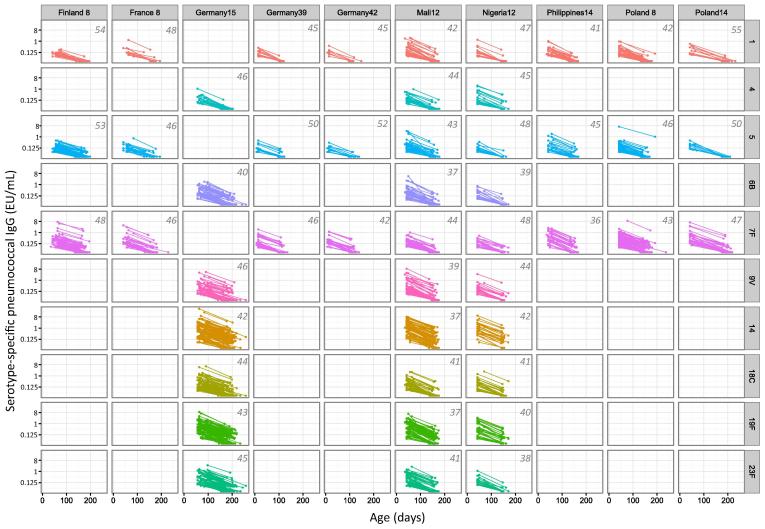
Serotype-specific pneumococcal antibody concentrations and estimated antibody half-lives in children with paired sera in 10 study cohorts. Top right corner of each panel displays the half-life (days) for each panel.

**Fig. 4A:**
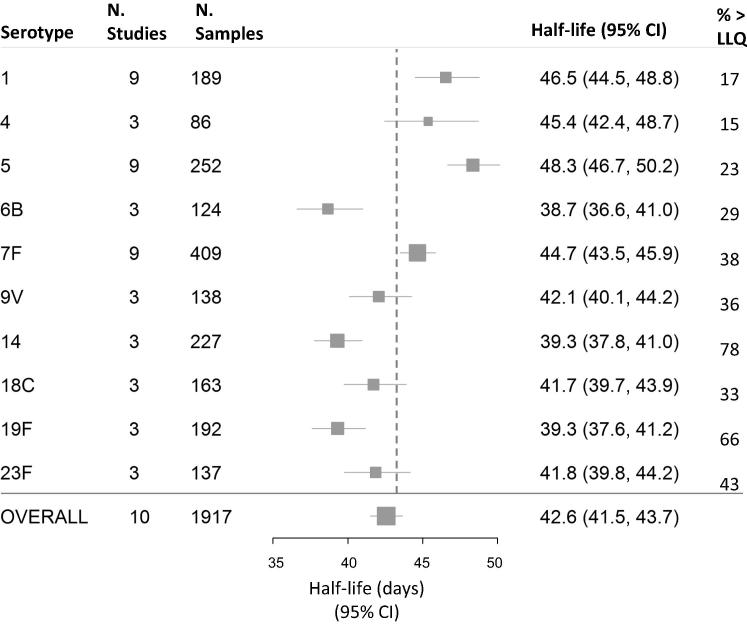
Individual participant data meta-analyses from mixed effects model of serotype-specific pneumococcal antibody half-lives. Heterogeneity across serotypes and country cohorts: F-test both p < 0.0001. % > LLQ: Percent of samples above the lower limit of quantification at the second time point. When weighted according to these percentages, the overall estimate was 41.5 days (95% CI 39.8–43.3).

There was statistically significant variation in half-life estimates between country cohorts and between serotypes (both p < 0.0001). Half-life estimates ranged from 38.7 days (95% CI 36.6–41.0) for serotype 6B, to 48.3 days (95% CI 46.7–50.2) for serotype 5. The overall estimate across serotypes was 42.6 days (95% CI 41.5–43.7) (Fig. 4A).

The timing of blood samples in the trials resulted in many samples being below the LLQ at the second time point. For these samples the time at which the antibody concentration fell below the LLQ is unknown and therefore the half-life is inflated. Those serotypes with high numbers of samples below the LLQ at the second time point were also the serotypes with higher estimated half-lives (Fig. 4A). When the overall half-life was estimated weighted according to the proportion of samples below the LLQ, the half-life was 41.5 (95% CI 39.8–43.3).

Results from the subgroup analysis of only PCV7 serotypes were very similar with statistically significant heterogeneity between serotypes and country cohorts (both p < 0.0001).

The age of the child was not significantly associated with decay rates (p = 0.103), confirming the assumption of exponential decay.

#### 3.2.2 Meningococcal antibodies

For the estimation of the half-lives of group A and C maternal meningococcal antibody, 502 pairs of samples from 344 infants were available (group A: 206 infants; group C: 296 infants) (Fig. 2B).

Half-lives were 43.1 days (95% CI 39.8–47.2) and 39.8 days (95% CI 33.4–49.4) for groups A and C respectively (Fig. 4B).

**Fig. 4B:**
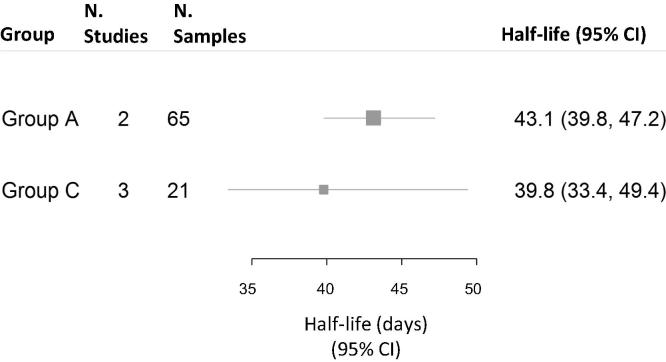
Individual participant data meta-analyses of capsular group-specific meningococcal antibody half-lives.

## 4 Discussion

Our analysis of 8022 infants reveals that in populations with no routine pneumococcal vaccine programme, substantial proportions of infants have antibodies to many vaccine serotypes of pneumococcus at the age when a vaccine programme might normally commence. Conversely, antibodies against capsular groups A and C meningococcal polysaccharides were less common, particularly for group C which was only present in 13% of infants in the four countries contained in this analysis. Higher levels of group A meningococcal antibodies than group C have also been seen in unvaccinated adults of childbearing age in the Netherlands [8], and in mothers in the UK [9]. Passively acquired maternal antibody has been shown to adversely affect the magnitude of the immune response to vaccination with pneumococcal conjugate vaccine [1], and increase the occurrence of otitis media in infants under 6 months of age [10].

The proportion of young infants with maternal anti-pneumococcal antibody differed between serotypes, with serotype 14 pneumococcal antibodies being present in almost all infants, and very high proportions of infants with serotype 19F antibodies. We have previously shown that the antibody response to vaccination with pneumococcal conjugate vaccine is adversely affected by the presence of maternal antibody. This inhibitory effect is greatest for serotype 14, with children seropositive from maternal antibodies having a response to vaccination that is only three-quarters the magnitude of those with no maternal antibody [1].

Antibody concentrations in infants are highly correlated with the concentration of serum antibody in mothers during pregnancy. However, antibody may also be transferred across the placenta during pregnancy at different rates. A higher rate of transplacental transmission for serotype 14 than for other serotypes has been reported in a study of 15 mother-infant pairs in Brazil [11] however this is not consistently seen [12]. In the larger study by Gupta et al. of HIV uninfected mother-infant pairs in Bangladesh, the geometric mean cord:maternal antibody ratio was similar across all serotypes with confidence intervals overlapping for all. Similarly, in studies of meningococcal vaccines in mother-infant pairs, similar rates of transplacental transfer have been observed for groups A and C meningococcal antibodies in both unvaccinated and vaccinated mothers [3], [13]. Differences in seroprevalence of antibodies between serotypes or between capsular groups in our analysis are therefore more likely to be due to serotype-specific differences in the magnitude of the antibody responses to nasopharyngeal carriage in mothers, and variation in the serotypes that circulate in any given community.

In our study, maternal pneumococcal antibody decayed exponentially with an average half-life of 42.6 days, however, variation between serotypes was significant. The half-lives for serotypes 6B, 14, and 19F maternal antibodies were 3 to 4 days shorter, whilst half-lives for serotypes 1, 4, 5, and 7F were between 2 and 6 days longer than the overall average. Previous studies have assumed the same rate of decay for passively-acquired antibody. Shahid et al. in a study in 55 Bangladeshi mother-infant pairs estimated a half-life for maternal pneumococcal antibodies of about 35 days based on serotypes 19F and 6B [2]. Antibodies against these two serotypes had similar half-lives of 39 days in our analysis thus the reason for the shorter overall half-life reported in that study may be due to the choice of serotypes against which antibodies were measured. O’Dempsey et al. describe a half-life of 20–30 days for antibodies against 6 serotypes of pneumococcus.[6]

The half-lives estimated for group A and C meningococcal antibodies of 43 and 40 days are comparable to the estimates for pneumococcal antibodies thus it is possible that passively-acquired anti-capsular antibodies for both species decay similarly. Similarly, Shahid et al. report a median half-life of 45 days [4]. However, O’Dempsey et al. describe a much lower half-life of 20 days for groups A and C maternal meningococcal antibodies [3]. It is unclear what factors may contribute to the much lower estimate in the O’Dempsey study, as the calculations are not described and the half-life is described as ‘approximate’. However, of note is that mothers in that study were vaccinated in pregnancy with a conjugate vaccine, whereas in our study, no maternal vaccination programmes were in place.

Differences in distribution of IgG subclasses [14], Fc structure/Fc receptor binding [15], and glycosylation [16] of maternal antibodies against the different polysaccharides could potentially explain the variation in half-lives observed in the current study. However, of note is that longer half-life estimates were observed for the serotypes and capsular groups with a higher proportion of data below the LLQ at the second time point. Thus bias due to the LLQ is a contributing factor to the variation observed.

Similar estimates to those seen in our meta-analysis have been reported for half-lives of other antigen-specific antibodies, including 36 days for antibodies against pertussis toxin and 40 days for filamentous haemaglutinin [7]. For anti-diphtheria toxin antibodies, estimates of 35 days and 31 days have been reported [5]. Longer half-lives have been reported for tetanus toxin (49–50 days [17]) and measles antibodies (46, 48, and 53 days) [18]. Due to the age of most studies which report half-lives of passively-acquired antibodies, only approximate estimates are provided, with no corresponding confidence intervals and no accounting for repeated measures.

These decay rates reported here for passively-acquired antibodies are in contrast to decay rates for antibodies induced by vaccination in infants. Vaccine-induced antibody decays rapidly in the first year after vaccination, after which time the rate of decay slows considerably [19].

Maternal prenatal vaccination programmes are currently recommended in many countries to protect infants against neonatal pertussis and tetanus. Other vaccines aimed at protecting infants through prenatal immunisation are currently in development, such as those for RSV and group B streptococcus, and may be followed by introduction of infant programmes as well. For pneumococcal disease, currently licensed infant conjugate vaccines are effective, but cover at most, 13 serotypes. Immunisation during pregnancy with the 23-valent polysaccharide vaccine, which is immunogenic in adults but not infants, may extend the number of serotypes against which infants are protected in the first months of life [20], [21], [22].

Implementation of a prenatal maternal immunisation programme covering the same antigens as an infant immunisation programme, gives rise to the potential for reduced vaccine responses in infants owing to higher levels of maternal passively-acquired antibody. Implementing complementary prenatal and infant programmes against the same antigens requires an understanding of the complex interplay between the influence of passive antibody on infant responses, and its rate of clearance from the body, in order to time such vaccination programmes effectively. Accurate modelling of the timing of infant immunisation doses therefore requires reliable estimates of antibody half-lives.

### 4.1 Strengths and limitations

The strength of this analysis lies in the large number of paired sera available from a wide range of countries and multiple antigens and serotypes. Samples which may skew estimates upwards due to the influence of carriage on antibody responses were excluded. Carriage of meningococcus is rare in young infants, however the influence of carriage cannot be ruled out entirely.

The trials from which these data derive were not conducted in order to estimate half-lives. Our substitution of the half the LLQ is an approximation which overestimates the half-life in these samples by at least 1 day. We chose not to exclude these participants due to the possibility that further bias would be introduced into the analysis by excluding a very large proportion of samples, including those with faster decay rates. Instead, the effect of the bias was estimated in a weighted sensitivity analysis.

Antibody half-lives estimated in this investigation are from populations with no routine programmes of immunisation in pregnancy, thus passively-acquired antibody is due to nasopharyngeal carriage or disease rather than immunisation and may differ from decay rates for passive antibodies induced by maternal vaccinations.

## 5 Conclusion

Substantial proportions of infants in a wide variety of countries have maternally-derived antibodies to many serotypes of pneumococcus at the age when a vaccine programme might normally commence, however few infants have maternally acquired antibodies to meningococcus. Maternally-derived passive antibody decays exponentially. Passively-acquired antibody decays with a half-life of 6 weeks, in line with previously described half-lives for antibodies against polysaccharide and protein antigens. Small but significant differences exist between serotypes or capsular groups, however these amount to differences of only a few days. These estimates are useful for modelling the impact of vaccination programmes that utilise a combination of both maternal and infant strategies, and consideration of schedules with a delayed start.
