# Supplementary material for: Prevalence and decay of maternal pneumococcal and meningococcal antibodies: A meta-analysis of type-specific decay rates
Source: Vaccine. 2017 Oct 13;35(43):5850–7. doi: 10.1016/j.vaccine.2017.09.002 (PMC5628610; doi:10.1016/j.vaccine.2017.09.002)
Supplement: Supplementary data 1 [file mmc1.docx]

# Supplementary Files

Table S1 Studies included in analyses of maternal pneumococcal antibodies

| Num | Study Number | Country | N  Infants | Age at enrolment (weeks), mean, [min, max] | Study vaccine schedule | Study title |
| --- | --- | --- | --- | --- | --- | --- |
| 8 | 105553 | Poland,  Finland,  France | 599,  835,  212 | 7.5 [5, 12]  10.0 [5, 12]  9.2 [6, 14] | 2, 3, 4  months | Assess lot-to-lot consistency of 3 lots (double blind design) of GlaxoSmithKline Biologicals' 10-valent pneumococcal vaccine and evaluate non-inferiority to Prevenar™ (single blind design) when administered as 3-dose primary immunization course before 6 months of age |
| 10 | 106208 | Chile | 235 | 8.3 [6, 12] | 2, 4, 6  months | To assess reactogenicity and immunogenicity of GSK Biologicals’ 10-valent pneumococcal conjugate vaccine, when co-administered with GSK Biologicals’ DTPa-HBV-IPV/Hib vaccine (Infanrix™ hexa) at 2, 4 and 6 months of age. |
| 12 | 110521 | Mali,  Nigeria | 233,  119 | 7.4 [5, 10]  6.3 [5, 9] | 6, 10, 14 weeks | Primary vaccination course in children receiving the pneumococcal vaccine GSK 1024850A co-administered with Zilbrix™ Hib and Polio Sabin™ |
| 14 | 107007 | Poland,  Philippines | 203,  200 | 7.4 [6, 12]  7.5 [5, 12] | 2, 4, 6 months, or  6,10,14 weeks | To assess the safety, reactogenicity and immunogenicity of GSK Biologicals' pneumococcal conjugate vaccine compared to Prevenar™, co-administered with DTPw-HBV/Hib & OPV or IPV vaccines as a 3-dose primary immunization course during the first 6 months of age |
| 15 | 217744/078 | Germany | 336 | 11.6 [8, 16] | 3, 4, 5  months | An open, multicentre, phase IV clinical trial to assess the immunogenicity and reactogenicity of GSK Biologicals’ combined DTPa-HBV-IPV/Hib vaccine, when co-administered at 3-4-5 Mth of age with Wyeth-Lederle's seven-valent pneumococcal conjugate vaccine at a different injection site during the same visit |
| 17 | 107017 | Czech Rep | 449 | 12.3 [8, 20] | 3, 4, 5  months | Multicentre study to assess the effect of prophylactic antipyretic treatment on the rate of febrile reactions following concomitant administration of GSK Biologicals’ 10-valent pneumococcal conjugate, Infanrix hexa and Rotarix vaccines |
| 18 | 107737 | Greece,  Spain | 63,  85 | 8.8 [8,10]  9.8 [7,16] | 2,4,6 months | Study to assess the safety and immunogenicity of GSK Biologicals 10-valent pneumococcal conjugate vaccine when Co-administered with DTPa-HBVIPV/ Hib (Infanrix-Hexa) vaccine in preterm infants as a 3-dose primary immunization course during the first 6 months of life. |
| 34 | 111188 | India | 360 | 1 month [1, 2 months] | 6, 10, 14 weeks | Primary vaccination course in healthy children receiving the pneumococcal vaccine GSK 1024850A co-administered with Tritanrix™-HepB/Hib at 6, 10 and 14 weeks of age |
| 39 | 105554 | Germany | 132 | 2.2 months [1, 4 months] | 2, 3, 4  months | Phase IIIa randomized, controlled study to assess the immunogenicity of GlaxoSmithKline (GSK) Biologicals’ 10-valent pneumococcal conjugate vaccine, when administered as a 3-dose primary immunization course before 6 months of age |
| 40 | 109563 | Japan | 359 | 13.5 [12, 16] | 3, 4, 5 months | Immunogenicity, safety and reactogenicity of GlaxoSmithKline Biologicals’ pneumococcal vaccine GSK1024850A following primary and booster vaccination of healthy Japanese children |
| 42 | 103488 | Germany | 677 | 2.1 months [1, 2 months] | 2, 3, 4 months | A randomized, controlled, phase II study to evaluate the safety and immunogenicity of different formulations of GlaxoSmithKline Biologicals’ 11-valent pneumococcal conjugate vaccine, when administered intramuscularly as a 3-dose primary immunization (2-3-4 month schedule) before 6 months of age |
| Total | 16 country cohorts | 13  countries | 5097  infants |  |  |  |

Table S2 Studies included in assessment of maternal meningococcal antibodies

| Num | Study Number | Country | No.  Infants | Age at enrolment (weeks), mean,  [min, max] | Study vaccine schedule | Study title |
| --- | --- | --- | --- | --- | --- | --- |
| 22 | 217744/076 | Spain | 467 | 9.1 [8, 12] | 2,4,6 months | An open, multicenter, phase IV clinical trial to assess the immunogenicity and reactogenicity of three doses of GSK Biologicals’ combined DTPa-HBV-IPV/Hib vaccine in healthy infants at 2, 4 and 6 months of age, when co-administered with Wyeth-Lederle’s meningococcal group C conjugate vaccine. |
| 27 | 217744/097 | Spain | 462 | 9.1 [7, 14] | 2,4,6 months | Study to assess immunogenicity and reactogenicity of three doses of GSK Bio’s combined Hib-MenC vaccine co-admind with GSK Bio’s DTPa-HBV-IPV vaccine and of two doses of Baxters meningococcal C conjugate vaccine co-admind with GSK Bio’s DTPa-HBV-IPV/Hib vaccine |
| 13 | 112157 | Poland | 411 | 8.6 [7, 12] | 2,3,4 months | Immunogenicity and safety study of GlaxoSmithKline Biologicals’ GSK2202083A vaccine in healthy infants at 2, 3 and 4 months of age |
| 43 | 100480 | Thailand | 850 | 8.5 [7, 11] | 2,4,6 months | Study to show lot-to-lot consistency of Hib-MenAC mixed with Tritanrix™-HBV, its non-inferiority to Tritanrix™-HBV/Hiberix™ with or without Meningitec™, and MenA response in 2, 4, 6 month infants with hepatitis B birth dose |
| 44 | 100478 | Philippines | 735 | 7.1 [5, 23] | 6,10,14 weeks | Study to show non-inferiority of Tritanrix™-HepB/Hib-MenAC (+/- hepatitis B vaccine at birth) versus Tritanrix™-HepB/Hiberix™ without hepatitis B vacc. at birth for antibody response to all vaccine antigens given in healthy infants |
| Total | 5 country cohorts | 4  countries | 2925  infants |  |  |  |
